# Development of a susceptibility gene based novel predictive model for the diagnosis of ulcerative colitis using random forest and artificial neural network

**DOI:** 10.18632/aging.103861

**Published:** 2020-10-24

**Authors:** Hanyang Li, Lijie Lai, Jun Shen

**Affiliations:** 1 Division of Gastroenterology and Hepatology, Key Laboratory of Gastroenterology and Hepatology, Ministry of Health, Inflammatory Bowel Disease Research Center, Shanghai 200127, China; 2 Renji Hospital, School of Medicine, Shanghai Jiao Tong University, Shanghai 200127, China; 3 Shanghai Institute of Digestive Disease, Shanghai 200127, China

**Keywords:** ulcerative colitis, predictive model, susceptibility genes, random forest, artificial neural network

## Abstract

Ulcerative colitis is a type of inflammatory bowel disease characterized by chronic and recurrent nonspecific inflammation of the intestinal tract. To find susceptibility genes and develop a novel predictive model of ulcerative colitis, two sets of cases and a control group containing the ulcerative colitis gene expression profile (training set GSE109142 and validation set GSE92415) were downloaded and used to identify differentially expressed genes. A total of 781 upregulated and 127 downregulated differentially expressed genes were identified in GSE109142. The random forest algorithm was introduced to determine 1 downregulated and 29 upregulated differentially expressed genes contributing highest to ulcerative colitis occurrence. Expression data of these 30 genes were transformed into gene expression scores, and an artificial neural network model was developed to calculate differentially expressed genes weights to ulcerative colitis. We established a universal molecular prognostic score (mPS) based on the expression data of the 30 genes and verified the mPS system with GSE92415. Prediction results agreed with that of an independent data set (ROC-AUC=0.9506/PR-AUC=0.9747). Our research creates a reliable predictive model for the diagnosis of ulcerative colitis, and provides an alternative marker panel for further research in disease early screening

## INTRODUCTION

Ulcerative colitis (UC) is a subtype of inflammatory bowel disease (IBD) characterized by chronic and recurrent nonspecific inflammation of the intestinal tract, manifesting as diffuse inflammation confined to the mucosa and submucosa of the large intestine [1]. The lesions of UC are commonly located in the sigmoid colon and rectum, and may extend to the entire colon. Over the past decade, UC prevalence is increasing worldwide, especially in developing countries. Ulcerative colitis can occur at any age, and majority of cases occur in late adolescence or early adulthood. Although no research has reported that the overall mortality rate in patients with UC is substantially different from that of the general population, UC leads to diminished quality of life with symptoms of abdominal pain, diarrhea, bloody mucopurulent stool, and autoimmune-related complications [2]. Therefore, the establishment of an accurate clinical prediction model (CPM) is of great importance to guide diagnosis and treatment.

Scientific and technological progress in the research field has contributed to several systematic sets of molecular prognostic indicator related CPMs of UC. The mRNA of neutrophil gelatinase-associated lipocalin (NGAL) showed overexpression in an inflamed intestine. Therefore, Budzynska et al. [3] used NGAL to predict the clinical and endoscopic activity of UC (area under curve [AUC] = 0.758). Hart et al. [4] combined fecal calprotectin (FC) with Mayo Endoscopic Score (MES) to predict endoscopic and histological activity of UC patients in clinical remission (AUC = 0.743). However, these predictive models are not efficient enough in the screening and early diagnosis of UC, given that clinical outcome assessments are based on a series of laboratory tests and operations. An effective and universal predictive model for early identification of and intervention in UC is lacking.

Although UC still remains unclear, abnormal immunoregulation, and genetic and environmental factors are thought to be relevant for UC pathogenesis [5]. Accumulating current evidence suggests that the pathogenesis of early UC involves the interaction of the susceptibility gene and environmental factors [6–8]. Similarity comparison between symptoms and gene functions has been used to predict pathogenic genes in IBD [9]. With the development of genome-wide association studies for UC, several validated susceptibility genes have been uncovered, including hMLH1, vitamin D receptor gene, etc. [10, 11]. Therefore, investigation of UC susceptibility genes is becoming the focus of UC screening and diagnosis.

Unsatisfying accuracy, low efficiency and loss of early screening prompt us to develop a novel UC predictive model. With the development of technology, machine learning has become a new approach in medical data processing. The random forest (RF) algorithm has been maturely applied in the field of Alzheimer’s disease and acute myeloid leukemia [12, 13]. Artificial neural network (ANN) also demonstrates powerful abilities in medical data processing [14]. Thus, our study aims to uncover UC susceptibility genes using RF and establish an ANN predictive model of UC. The predictive model can be used to provide early screening markers for diagnosis and treatment of UC.

## RESULTS

**Figure 1:**
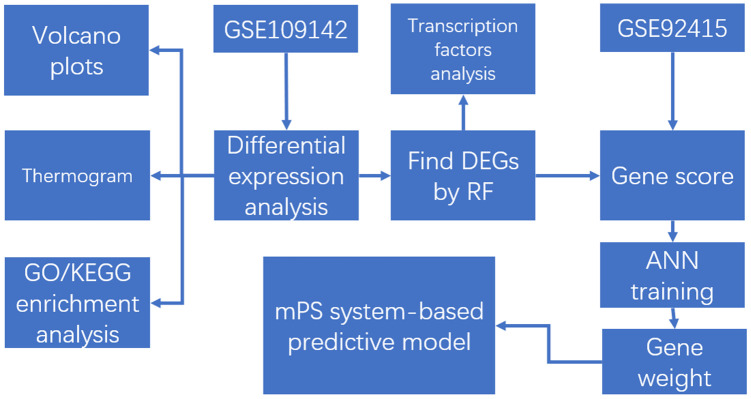
**The flow chart in the study. DEGs analysis, gene set enrichment analysis, machine learning, and construction and validation of the mPS system-based predictive model.**

The steps to our cohort study are listed in Figure 1, and information for the training dataset (GSE109142) and validation set (GSE92415) related to UC is listed in Table 1.

**Table 1:** The information of training/validation sets (GSE109142/GSE92415).

**Dataset ID**	**Platform**	**Ulcerative Colitis**	**Normal**	**Total**
GSE109142	GPL16791	206	20	226
GSE92415	GPL13158	53	21	74

### Screening of DEGs

**Figure 2:**
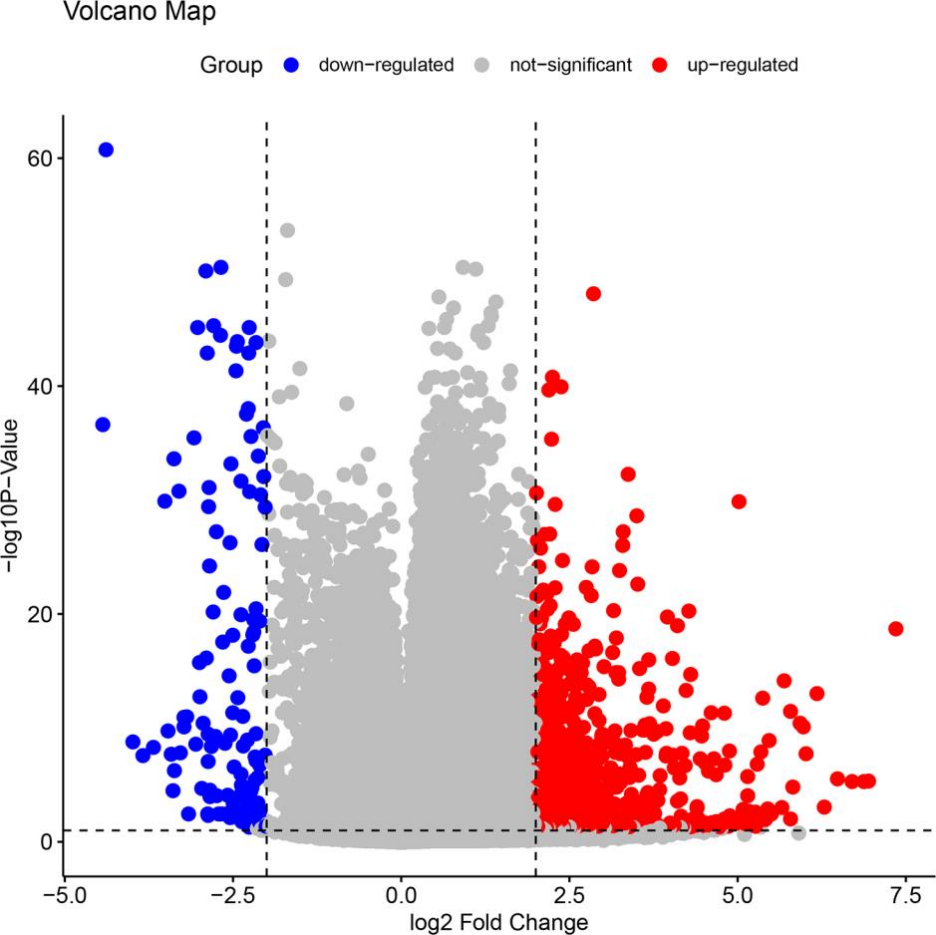
**The volcano plots of all the DEGs in GSE109142. In the map, each blue spot represents a down-regulated gene, whereas each red spot represents an up-regulated gene.** A clear demarcation can be identified between up-regulated genes and down-regulated genes.

**Figure 3:**
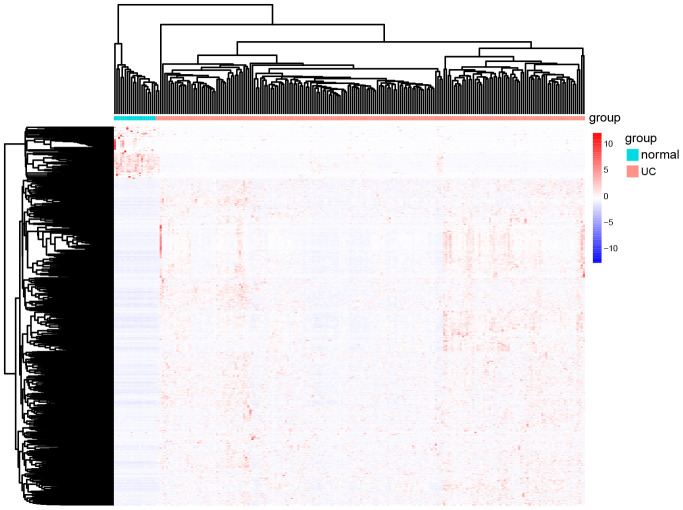
**The heat map of all the DEGs in GSE109142.** In the map, each list represents a gene and up-regulated genes and down-regulated genes also have a clear demarcation. Green/pink columns represents controls/cases (normal people/ UC patients).

The volcano map in Figure 2 shows the expression status of all DEGs in GSE109142. A clear demarcation can be identified between upregulated genes and downregulated genes. The heat map in Figure 3 shows the expression status of 908 DEGs, from which we observe the cases with higher levels of upregulated gene expression in comparison to the controls.

### DEG enrichment analysis

**Figure 4:**
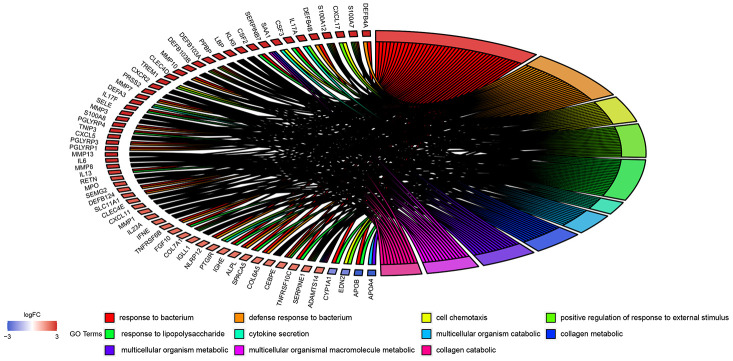
**GO enrichment analysis of 908 DEGs.** Genes are listed at the left side. Up-regulated genes are in red color, and down-regulated genes are in blue color conversely. The ligated bands between left and right side indicate that DEGs are related to the GO terms.

**Figure 5:**
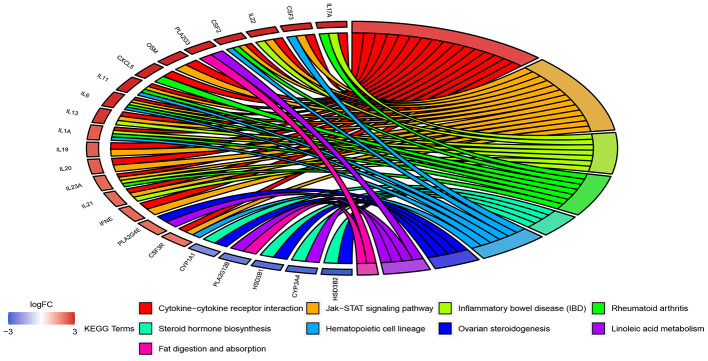
**KEGG pathway enrichment analysis of 908 DEGs.** The interpretation of Figure 5 is the same as Figure 4.

The 908 DEGs underwent Gene Ontology (GO) and Kyoto Encyclopedia of Genes and Genomes (KEGG) enrichment analysis. Results of GO analysis (p-value cutoff = 0.01) suggested that DEGs were significantly enriched in bacterial response-related biological processes, such as “response to bacterium” and “defense response to bacterium”. KEGG pathway analysis (p-value cutoff = 0.05) suggested that the DEGs were primarily involved in “cytokine–cytokine receptor interaction” and the “Jak-STAT signaling pathway” (Figures 4 and 5).

### Diagnosis-related DEGs with RF

**Figure 6:**
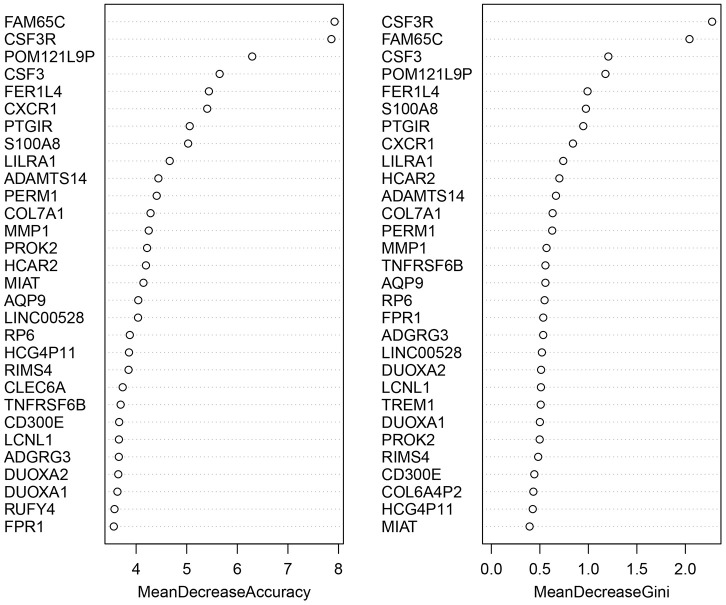
**The screening results of the top-30 UC-related DEGs by random forest classifier.** All the genes are sorted by the value of “Mean Decrease Accuracy” and “Mean Decrease Gini”. The greater the two values are, the closer relationship with UC the DEG has.

**Figure 7:**
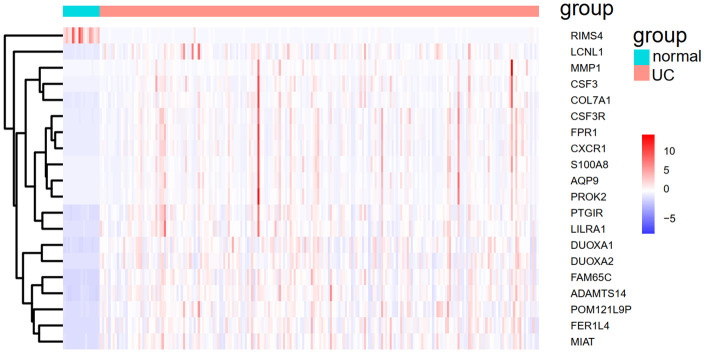
**The heat map of the top-30 UC related DEGs in GSE109142.**

We applied the expression data of 908 DEGs to a machine learning algorithm called RF classifier. The RF screening results are shown in Figure 6. From these sheets, we are able to identify the top-5 UC related genes in the 30 DEGs, including *FAM65C*, *CSF3R*, *CSF3*, *POM121L9P*, and *FER1L4*. The heat map showing the expression status of the top-30 DEGs can be seen in Figure 7.

**Figure 8:**
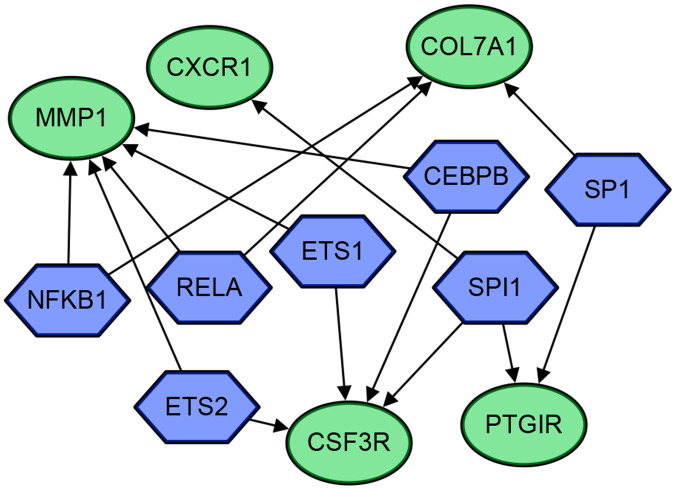
**The gene regulatory network diagram of the 5 genes and 7 transcriptional regulators.** The blue hexagons are transcriptional regulators, and the green circles are genes. The arrows indicate their regulatory relationships.

Thereafter, the 30 DEGs were put into the TRRUST web server. Seven transcriptional regulators (SPI1, ETS2, CEBPB, ETS1, RELA, NFKB1, SP1) were identified to be related with five DEGs (*MMP1*, *CXCR1*, *COL7A1*, *CSF3R*, *PTGIR*). The gene regulatory network diagram is shown as Figure 8 and Table 2.

**Table 2:** The relationships between genes and transcriptional regulators predicted by TRRUST.

**#**	**Key TF**	**# of overlapped genes**	**P value**	**Q value**	**List of overlapped genes**
1	SPI1	3	0.0000497	0.000348	CXCR1, CSF3R, PTGIR
2	ETS2	2	0.00063	0.00221	MMP1, CSF3R
3	CEBPB	2	0.0022	0.00514	MMP1, CSF3R
4	ETS1	2	0.00379	0.00663	MMP1, CSF3R
5	RELA	2	0.0475	0.0561	MMP1, COL7A1
6	NFKB1	2	0.0481	0.0561	MMP1, COL7A1
7	SP1	2	0.104	0.104	COL7A1, PTGIR

### ANN-based establishment of molecular prognostic score

With expression data transformation of 30 DEGs into “Gene_Score”, the weight of each gene was optimized with a neural network algorithm (Table 3). The mPS was calculated by summation of “Gene_Score” × “Gene_Weight” for all 30 DEGs [15]. Thereafter, we regarded the mPS of 226 samples as predicted values, and the clinical outcomes of UC as true values. Following calculation in the ROCR package in R version 3.5.3, the ROC-AUC of our predictive model was 0.9847, and the PR-AUC of our predictive model was 0.9444.

**Table 3:** The “Gene_Weight” of 30 DEGs in GSE109142.

**Gene symbol**	**Gene_Weight**
DUOXA2	3.1869
S100A8	3.0181
TNFRSF6B	3.7603
MMP1	4.5608
CSF3R	3.0028
FER1L4	3.0258
COL7A1	3.6774
FPR1	3.0121
FAM65C	3.0217
AQP9	3.0115
MIAT	3.0328
PROK2	3.0119
RP6	4.5567
CSF3	4.0558
ADGRG3	3.0259
HCAR2	3.1662
CXCR1	3.0118
ADAMTS14	4.3368
PTGIR	3.0149
DUOXA1	3.2243
CD300E	4.3764
LILRA1	3.1524
HCG4P11	1.1187
LINC00528	3.7521
PERM1	4.2631
RUFY4	4.7713
POM121L9P	4.6224
LCNL1	4.5594
CLEC6A	3.0244
RIMS4	4.7128

### Validation of UC predictive model

**Figure 9:**
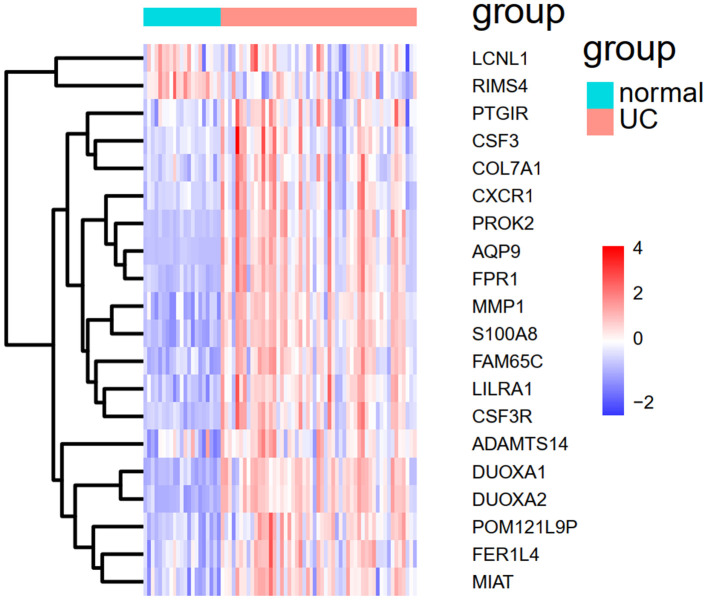
**The heat map of the 20 overlapped genes between GSE109142 and GSE92415.**

To demonstrate whether the mPS system can predict the occurrence of UC, not only in the training set but also in other independent UC cohorts, we introduced an independent UC dataset (GSE92415) to validate the model. The heat map of 20 overlapped genes between GSE109142 and GSE92415 is shown in Figure 9. Similarly, the expression status of the 20 genes was transformed into a “Gene_Score” sheet containing 74 lines of samples, 20 columns of DEGs and 1 column of UC outcome variable (case/control). The “Gene_Weight” and mPS were calculated in the same way as GSE109142. Thereafter, we regarded the mPS of 74 samples as predicted values, and the clinical outcomes of UC as true values. The ROC-AUC of the predictive model in GSE92415 was 0.9506, and the PR-AUC was 0.9747.

## DISCUSSION

UC has first emerged as a problem in industrialized countries and continued to rise in developing areas [16]. Besides the considerable cost, patients with active ulcerative colitis present high rates of fatigue, inferior health-related quality of life, and high disability [17]. Data also supports an association between UC and colorectal cancer [18]. Therefore, the necessity for UC management will be greater than ever. A prediction model based on valuable indices is intuitive and easy to use, which will facilitate decision making in clinical practice. In our study, we established an integrated toolkit of disease occurrence-related genes for UC, and developed a combination of machine learning algorithms to introduce a new clinical prediction score named mPS that is suitable for general UC patients. As a compatible and efficient scoring system, mPS proved useful in the evaluation of patient subpopulations in an independent platform.

The GO and KEGG enrichment analysis suggest that UC-related DEGs are involved in diversified GO terms and pathways, which reflects the dynamics and complexity of its pathogenesis. In practice, a link between alteration of intestinal flora and the occurrence of UC has been suggested previously [19]. Moreover, we observed that bacterial response-related biological processes are most prominent in GO terms. The best characterized pathway among the whole KEGG pathway is “cytokine–cytokine receptor interaction.” As primary mediators of mucosal healing in IBD, cytokines including interleukin (IL)-1, IL-6, IL-10, and IL-13 occupy an important position in the Th2 atypical immune response of UC [20]. A marked difference in the levels of IL-4, IL-8, and IL-17A were also reported among UC patients and control patients [21]. The correct interpretation of GO and KEGG enrichment analysis results supports the research of DEGs and their related metabolic pathways, leading to the discovery of new diagnostic indicators and therapeutic targets.

TRRUST analysis identified seven transcriptional regulators (SPI1, ETS2, CEBPB, ETS1, RELA, NFKB1, SP1) related to five DEGs (MMP1, CXCR1, COL7A1, CSF3R, PTGIR). Although it has been proved that the NFKB1 promoter polymorphism is not associated with UC [22, 23], the correlation between transcriptional regulators and DEGs are still worth exploring in further studies.

The innovative combination of machine learning approaches is the highlighted novelty of our study and has been used to improve the predictive ability of our UC predictive model, which has achieved good results in predictive ability creatively. As a form of an ensemble algorithm, RF has an outstanding performance on the processing of multiple-featured data with high accuracy and precision. The RF algorithm has been widely applied with success in the detection and prediction of clinical diseases such as Type 2 diabetes (AUC=0.89) and metabolic syndrome (accuracy>98%) [24, 25]. The ANN model has also been developed to predict the severity of IBD based on meteorological data and achieved high accuracy because of its self-learning ability and high efficiency [26]. Particularly, the cooperative machine learning approaches of RF and ANN was were reported to be efficient in many several data generation processes, including those with transcriptome profiles [27]. The development of mPS system has also been proved to be simple and cost-effective in the prediction of overall survival of brest cancer patients [15]. However, corresponding predictive models have not been used to predict the clinical outcome of UC patients. Previously, several systematic sets of molecular prognostic indicator related predictive models of UC have been established. Based on mRNA overexpression of NGAL in an inflamed intestine, Budzynska et al. used NGAL to predict the clinical and endoscopic activity of UC (AUC = 0.758) [3]. In a similar manner, Hart et al. combined FC with MES to predict endoscopic and histological activity of UC patients in clinical remission (AUC = 0.743) [4]. The mPS-based predictive model in our study shows higher overall accuracy and precision in both the training set and validation set (AUC = 0.9847 in GSE109142 and AUC = 0.9506 in GSE92415 respectively). In contrast to the two predictive models, machine learning and processing patterns are more reliable and accurate in data analysis. As data resources, we used downloaded UC gene expression profiles containing cases and controls to lower subjective errors during the calculation of the predictive model, such as the subjective nature of MES. Alternatively, the collection of UC gene expression profiles is much easier and more cost- effective than that of clinical patient information. With the AUC of the predictive model achieving 0.9506 in the independent validation set (GSE92415), the general applicability of the mPS system is also confirmed. RF classifier screening results identified CSF3R as the best-characterized DEG among all UC occurrence-related genes. It is worth noting that the upregulation of CSF3R is closely related to macrophage recruitment, which is regarded as a hallmark of IBD [28]. Therefore, investigations on the application of the mPS system in Crohn’s disease is expected in future work.

However, our study still has several limitations. Firstly, numerous environmental factors have been linked with UC and may either increase the risk of or protect against developing this condition and can also become modifiers of UC course [29]. Our susceptibility gene based novel predictive model may has limited value in predictive power as others to some extent. Secondly, though we have validated the UC-related DEGs in the large expression profile GSE109142, the validation set GSE92415 is relatively small. The mPS system development and the predictive model was based on the dataset from the GEO, thus our predictive model should be practiced and verified in laboratory experiments and clinical work. In fact, there are a variety of protocols for the preservation of UC samples, which may present challenges to our original intention of building a “platform-independent” score system suitable to data acquired from any method. Comparison of different protocols and establishment of a universal and accurate method for the detection of the expression level of the 30 DEGs are still challenges that must be overcome before the application of the mPS system in clinical trials. Indeed, all analyses in our study were conducted in a retrospective manner. Regarding methodological rigor, prospective studies are also indispensable to validate our findings. We believe that the development of the mPS-based predictive model can not only optimize the screening and early diagnosis of UC, but also prompt further biochemical research and provide new ideas for its clinical treatment.

## MATERIALS AND METHODS

### Study design and process

We downloaded two sets of UC gene expression profiles containing cases and controls (GSE109142 and GSE92415). Both sets are independent UC cohorts used multiple times and have proved to be accurate and reliable in recent UC-related research. The *t* test package in R version 3.5.3 was used to screen DEGs between cases and controls according to their specific threshold. Further analysis of DEGs included volcano plots, heat maps, gene ontology (GO) functions and KEGG pathway enrichment analyses. The RF algorithm was introduced to determine DEGs contributing the highest occurrence of UC. The analysis of transcription factors related to the regulation of these DEGs was also performed. Expression data of DEGs from all samples were transformed into gene expression scores. Based on these, we developed an ANN model to calculate the respective weight of DEGs to UC. Thereafter, we established an mPS system based on the expression data of these DEGs and verified it with GSE92415 to evaluate the accuracy of our predictive model.

### Data resources

GSE109142 (http://www.ncbi.nlm.nih.gov/geo/query/acc.cgi?acc=GSE109142) and GSE92415 (http://www.ncbi.nlm.nih.gov/geo/query/acc.cgi?acc=GSE92415) were downloaded from the GEO database (Table 1). The data preprocessing was implemented as follows. The expression estimates in GSE109142 were measured in transcripts per million. The expression estimates in GSE92415 were measured in log2-transformed quantile-normalized signal intensity. We mapped probes to genes and removed the unloaded probes. If more than one probe corresponded to a certain gene, we considered the median probe value as the final expression value of the specific gene.

### Screening for DEGs

The t test package in R version 3.5.3 was used to identify DEGs of GSE109142. We performed the analysis with set threshold: |log2(FC)| >2 and Benjamini-Hochberg adjusted p < 0.05. Finally, we gotacquired a list of 908 DEGs, including 781 upregulated DEGs and 127 downregulated DEGs.

### Volcano plots and heat map

Volcano plots of all the genes in GSE109142 were drawn byusing the ggpubr package ("ggplot2" based publication ready plots) in R version 3.5.3. The heat map of DEGs in GSE109142 was drawn byusing the pheatmap package in R version 3.5.3. Among the DEGs, the red color indicatesd upregulation, and the blue color indicatesed downregulation.

### GO function and KEGG pathway enrichment analysis

GO and KEGG pathway enrichment analysis were used to identify the functions of DEGs related to UC in GSE109142. These were performed on the DAVID website (http://david.abcc.ncifcrf.gov). The GOplot package in R version 3.5.3 was used for the visualization of the results.

### Screening of diagnosis-related DEGs

RF is a general technique for the training and prediction of samples based on the classification tree. In our study, the randomForest package in R version 3.5.3 was used to determine the top-30 DEGs according to their contribution to the occurrence of UC. The screening threshold was set as mean decrease of accuracy ≥ 3.56 and mean decrease of gini index > 0.53. In this manner, the top-30 DEGs were analyzed on the TRRUST web server (https://www.grnpedia.org/trrust/) to find related transcriptional regulators that can be used to explain observed gene expression changes and identify potential signaling pathways involved in the pathogenesis of UC.

### Calculation of gene_weight using ANN

The expression data of the 30 DEGs were first transformed into “Gene_Score” based on their expression level. In the case of a certain sample, the expression value of a specific gene was compared to the median of all sample expression values of that sample. If the expression value of the upregulated gene is higher, it will be valued as 1, otherwise 0. Similarly, if the expression value of downregulated gene is higher, it will be valued as 0, otherwise 1. The outcome variable was the occurrence of UC: cases were valued as 1, controls were valued as 0. Finally, we received a “Gene_Score” sheet containing 226 lines of samples, 30 columns of DEGs and 1 column of UC outcome variable (case/control).

The establishment of ANN was achieved by the Keras package in R version 3.5.3 (UC outcome variable = y, the expression values of DEGs = x). The rectified linear unit (RELU) was used as an activation function for thr hidden layers [30, 31]; correspondingly, softmax functions were used for the output layers [32]. Cross entropy was used as the error function [33]. Adam algorithm was introduced to optimize each “Gene_weight,” and speed up training [34]. Following ANN training, the “Gene_Weight” of a specific DEG was valued as the maximum weight of that DEG in the hidden layer [15].

### Construction and validation of the UC predictive model

The mPS scoring system played an important role in the construction of the UC predictive model. As a new type of scoring system, mPS has obtained success in precise prediction of overall survival of breast cancer patients [15]. “Gene_weight” of a specific DEG was achieved with the help of a combination of RF and ANN. The mPS of a certain sample was calculated by summation of “Gene_Score” × “Gene_Weight” for all 30 DEGs [15].

The independent dataset GSE92415 was used for the validation of the mPS scoring system based on GSE109142. We took the intersection of the 30 DEGs in GSE109142 and all genes in GSE92415 to obtain final lists of 20 overlapped genes. Within the intersection, the expression levels of 20 genes were transformed to binary status (above the median or below the median). As follows, “Gene_Score” and “Gene_weight” were valued according to the data processing methods mentioned above. The mPS of the validation set was also calculated by the summation of “Gene_Score” × “Gene_Weight”. The ROCR package in R version 3.5.3 was used to calculate the AUC, which was regarded as an indicator to evaluate the predictive performance of our model.
